# Auxin-independent depletion of degron-tagged proteins by TIR1

**DOI:** 10.17912/micropub.biology.000213

**Published:** 2020-01-28

**Authors:** Erin C Schiksnis, Angela L Nicholson, Matthew S Modena, Makena N Pule, Joshua A Arribere, Amy E Pasquinelli

**Affiliations:** 1 Division of Biology, University of California, San Diego, La Jolla, CA; 2 Department of MCD Biology, University of California, Santa Cruz, Santa Cruz, CA

**Figure 1:**
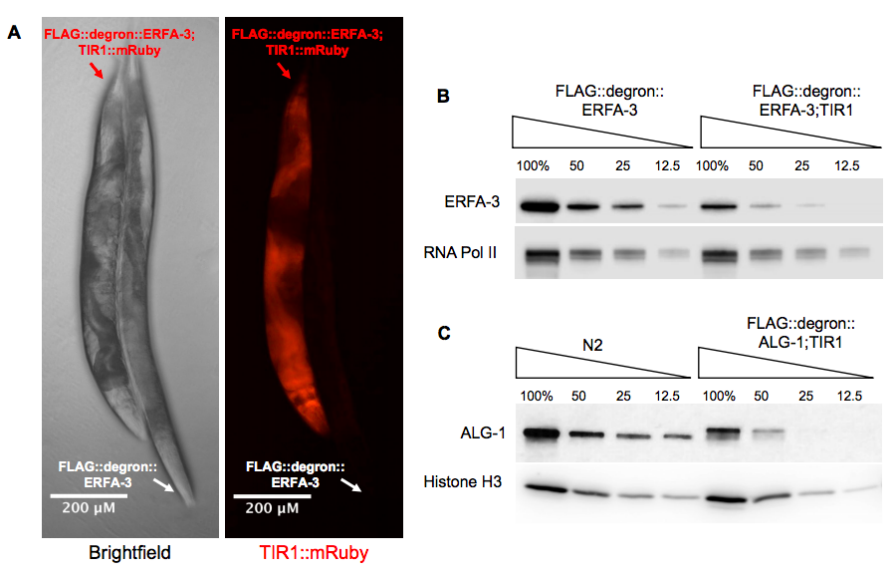
A. Example side-by-side micrograph of FLAG::degron::ERFA-3 and degron::ERFA-3;TIR1 adult animals. Expression of TIR1 tagged with mRuby was visualized by fluorescence microscopy. The shorter and thicker appearance of the TIR1 expressing strain is characteristic of the dumpy phenotype. B. Western blot of ERFA-3 protein levels in FLAG::degron::ERFA-3 and FLAG::degron::ERFA-3; TIR1 mixed-stage animals cultured at 15°C. C. Western blot of ALG-1 protein levels in N2 and FLAG::degron::ALG-1; TIR1 L4 stage animals cultured at 20°C.

## Description

The auxin inducible degron (AID) system was developed to enable conditional depletion of proteins of interest. In this system, a plant-derived F-box protein, TIR1, is expressed and complexes with endogenous Skp1 and Cullin proteins to form an E3 ubiquitin ligase complex. Proteins of interest can be tagged with a plant-derived degron, which is recognized by the TIR1-ubiquitin ligase complex in the presence of the plant hormone auxin (indole-3-acetic acid) (Nishimura et al. 2009). This system was recently adapted for *Caenorhabditis elegans,* wherein TIR1 constitutive expression enables rapid depletion of degron-tagged proteins of interest when animals are transferred to NGM plates supplemented with auxin. In *C. elegans,* the AID system has allowed for fine-tuned spatiotemporal control of protein expression and has been used to deplete many essential proteins, which are difficult to study using classical genetic approaches (Zhang et al. 2015). Here, we highlight a limitation of this system by demonstrating that TIR1 expression can cause depletion of degron-tagged proteins without auxin addition. These results corroborate observations from human cell lines, where TIR1 has been shown to act independently of auxin to chronically deplete degron-tagged proteins (Sathyan et al. 2019).

In an attempt to study the effects of depletion of the essential protein ERFA-3, we used CRISPR-Cas9 to insert a degron sequence at its N-terminus. The resulting *3xflag::degron::erfa-3* strain (WJA2126), was backcrossed three times to N2 and then outcrossed to CA1200, a publicly available pan-somatic *TIR1::mRuby* strain (*ieSi57 [eft-3p::TIR1::mRuby::unc-54(3’UTR) + Cbr-unc-119(+)] II*), to make a new strain, PQ631. Expression of TIR1 in the *3xflag::degron::erfa-3* strain led to gross morphological defects that are not seen in the *3xflag::degron::erfa-3* or TIR1-expressing strains (Zhang et al. 2015) (Figure 1A). The *3xflag::degron::erfa-3; ieSi57 [Peft-3::TIR1::mRuby::unc-54 3’UTR, cb-unc-119(+)] II* strain exhibited bagging and dumpy phenotypes as well as fertility defects that manifested as an observed slower growth rate of the overall population in comparison to its parental strains. Individuals of this strain tended to lay fewer viable eggs than control strains. It is yet unclear whether this is due to defects in meiosis, embryogenesis, or in any of the numerous steps in-between. To understand the cause of these phenotypic abnormalities, we performed a quantitative Western blot against ERFA-3 using an anti-FLAG antibody (Figure 1B). Here, it is evident that expression of TIR1 alone, despite no addition of auxin to the media, leads to depletion of 3xFLAG::degron::ERFA-3 to approximately 30% of the levels observed in the absence of TIR1.

We observed a similar phenomenon with the *alg-1* locus. We analyzed QK155 (*4xflag::degron::alg-1; ieSi57 [eft-3p::TIR1::mRuby::unc-54(3’UTR) + Cbr-unc-119(+)] II*). This strain expresses degron-tagged ALG-1 and pan-somatic TIR1. We performed a quantitative Western blot to compare ALG-1 expression in our 4xFLAG::degron::ALG-1; TIR1 strain to N2 using an antibody against endogenous ALG-1. As with ERFA-3, degron-tagged ALG-1 was chronically depleted when TIR1 was expressed pan-somatically. The ALG-1 protein level in the 4xFLAG::degron::ALG-1;TIR1 strain was approximately 25% that of endogenous protein expression (Figure 1C).

Taken together, these results indicate that TIR1 can act without the addition of auxin to the media, ultimately resulting in chronic depletion of degron-tagged proteins. There are at least two, non-mutually exclusive possibilities to explain our observations: (1) Auxin may exist in one or more components of the *C. elegans* media used. Seaweed is a common source of agar and does contain some auxin, though it is unclear if the concentration would be sufficient to activate TIR1. (2) There may be auxin-independent recognition of the degron by TIR1. Regardless of the mechanism of our observation, the effects were observed in at least two separate labs over the span of several months, indicating it is a potentially widespread caveat of the approach.

The phenotypes we observed for the *3xflag::degron::erfa-3* strain in the presence of TIR1 are consistent with published phenotypes associated with RNAi knockdown of *erfa-3,* which include sterility, slow development, and a high rate of embryonic lethality (Kamath et al. 2003). Yet, when the AID system was adapted for *C. elegans*, various phenotypic assays were performed to demonstrate that TIR1 expression, with multiple different degron-tagged essential proteins, resulted in no observable abnormalities (Zhang et al. 2015). This suggests that not all degron-tagged proteins may be subject to auxin-independent degradation by TIR1 or that chronically lower levels of some essential proteins can be tolerated. In conclusion, our results highlight the need to carefully analyze expression levels of degron-tagged proteins of interest as they may be subject to TIR1-mediated degradation prior to auxin exposure.

## Methods

**Imaging:** WJA2126 and PQ631 animals were cultured at 15°C and grown until adulthood before being anesthetized with 1 mg/ ml of Levamisole and imaged at 10X magnification. Imaging was performed with Zeiss Axio Imager.A1.**Western blotting:** Western blotting was performed as previously described (Zisoulis et al. 2010; Van Wynsberghe et al. 2011), using mouse monoclonal antibodies against FLAG (Sigma) and RNA polymerase II (Covance) or rabbit polyclonal antibodies against Histone H3 (Abcam) and ALG-1 (ThermoFisher Scientific).

## Reagents

**Nematode culture:** *C*. *elegans* strains were cultured under standard conditions and synchronized by hypochlorite treatment (Wood 1988).**Strain construction:** *erfa-3* was tagged with *3xflag::degron* by CRISPR/Cas9 using the co-conversion strategy as previously described (Arribere et al. 2014) using overlapping ultramers (IDT) encoding the tag as outlined previously (Paix et al. 2016). Integration at the *erfa-3* locus was confirmed by sequencing. The sequence of the insertion with flanking homologies is aagcgatttcagagctctcggcatcgacgcaaaATGTCCgattataaagatcatgacggagattataaagaccatgatattgatt ataaagatgacgatgataagATGCCTAAAGATCCAGCCAAACCTCCGGCCAAGGCACAAGTTGTGGGATGGCCACCGGTGAGATC ATACCGGAAGAACGTGATGGTTTCCTGCCAAAAATCAAGCGGTGGCCCGGAGGCGGCGGCGTTCGTGAAGTCAGGCTGGAACGTG AACGCCTCGTCGTTTGTGCCAAA.**Strains:**

WJA2126: *erfa-3(srf2126 | 3xflag::degron::erfa-3) V*, this study

CA1200: *ieSi57 [eft-3p::TIR1::mRuby::unc-54(3’UTR) + Cbr-unc-119(+)] II*, CGC (The *eft-3* gene is also referred to as *eef-1A.1*.)

PQ631: *erfa-3(srf2126) V; ieSi57 II*, this study

QK155: *alg-1(xk20 | 4xflag::degron::alg-1) X; ieSi57 II*, gift from the John Kim Lab
